# General movement based therapy to support neurodevelopment of preterm infants: a randomized clinical trial

**DOI:** 10.1038/s41390-025-04734-2

**Published:** 2026-01-09

**Authors:** Anna Badura, Annika Dietz, Florian Zeman, Sarah Klarner, Luisa Ammon, Maria Waltner-Romen, Elke Griesmaier, Maike Wellmann, Verena Lehnerer, Sven Wellmann

**Affiliations:** 1 Department of Neonatology, University Children’s Hospital Regensburg (KUNO), Hospital St. Hedwig of the Order of St. John of God, University of Regensburg, Regensburg, Germany; 2 Center for Clinical Studies, University Hospital of Regensburg, Regensburg, Germany; 3 Department of Pediatrics II (Neonatology), Medical University of Innsbruck, Innsbruck, Austria

## Abstract

**Background:**

Preterm birth increases the risk of neurodevelopmental impairments, emphasizing the need for early interventions. This study aimed to assess the feasibility and effectiveness of a General Movement (GM)-based intervention on infant neurodevelopment and parental mental health.

**Method:**

In a prospective, randomized-controlled trial, very preterm infants (gestational age <32 weeks or birth weight <1500 g) were enrolled between October 1, 2021, and June 6, 2023. Infants received a three times daily GM-based treatment by trained parents over 10 weeks starting at 34 weeks PMA or standard care. Primary outcome was neurodevelopment until 2 years’ corrected age, secondary outcomes included parental mental health and serum levels of brain damage biomarkers.

**Results:**

Sixty-six infants were randomized (32 control, 34 intervention). The median birth weight was 1243 g (IQR, 919-1623 g) in the control group and 1035 g (IQR, 853-1230 g) in the GM group. No significant group differences were observed for neurodevelopment outcome and parental mental health. Interestingly, all three infants displaying poor neuromotor features in the intervention group before treatment showed good neurodevelopment in the follow-up.

**Conclusion:**

Our findings suggest a potential role of GM-based intervention in high-risk preterm infants. Future research should focus on improved participant selection and adherence.

**Impact:**

- A General Movement (GM)-based early intervention starting at 34 weeks PMA, led by parents with telehealth support over 10 weeks from pediatric physiotherapists, was both feasible and well-received.
- Infant neurodevelopment until 2 years’ corrected age and parental mental health were similar in both the intervention and control groups.
- The approach may be especially helpful for preterm infants who show early signs of neurodevelopmental challenges.
- As one of the first studies of its kind, this RCT adds valuable knowledge about GM-based therapy for very preterm infants.
- The results support the importance of personalized early interventions to meet the unique needs of each infant.

## Introduction

Preterm birth (<37 weeks’ gestation) accounts for approximately 10% of all live births worldwide, with about 1% of infants being born very prematurely (<32 weeks’ gestation).^1^ While survival for preterm infants have steadily increased over recent decades, neurodevelopment did not.^2^ About 15–20% of very preterm infants show impaired cognitive or motor development by the age of 2 years, often experiencing ongoing cognitive, emotional, and social challenges into childhood and young adulthood.^3,4^

Early interventions and rehabilitative therapies may help prevent dysmaturation of white and gray matter structures in preterm infants, whether directly or indirectly related to brain injuries in preterm infants.^5^ Several studies have identified concepts, factors and supportive early interventions aimed to improve preterm developmental outcome.^6–8^ Among them, motor interventions comprise a specific group but it is unclear which kind of motor interventions are effective, at which time point they should be commenced, if a start during NICU stay is beneficial, and what the role of parents is.^9–13^

Fetal movements are crucial for motor and cognitive development.^14^ They are known as general movements (GM) and occur during fetal and infant development until 3–5 months of corrected age (CA)^15^ and GM assessment supports diagnostic in preterm infants neuromotor development.^16^ GMs are generated by the Central Pattern Generator (CPG) network, the activity of which is modulated by supraspinal projections and sensory feedback.^17^ It has been supposed that imitating these movements after birth may provide a therapeutic intervention in compromised preterm infants.^18^ Soloveichick’s group initiated an early intervention approach conducted by therapists with imitation of age-specific GM patterns, Movement Imitation Therapy for Preterm Babies (MIT-PB).^18^ The concept behind this MIT-PB approach is that the CPG network can be modulated by increasing the variability of sensory feedback through variable movements of the extremities. To date, this therapeutic approach has only been tested in a few infants and resulted in positive outcomes.^18^

Building upon these initial promising findings and consistent with recent evidence indicating that parent-delivered interventions may be more effective than therapist-administered therapies,^13^ we refined the approach initially established by Soloveichick’s group. In addition, we incorporated selected music therapy into the intervention,^19^ hypothesizing that all very preterm infants, regardless of cerebral comorbidities, could benefit from enhanced, diverse, and meaningful sensory input in the often sterile and unnatural NICU environment.

To evaluate the feasibility and efficacy of this GM-based intervention, we conducted a randomized clinical trial (RCT) in very preterm infants with outcomes assessing both infant neurodevelopment and parental well-being. Neurodevelopmental outcomes for infants were evaluated at 3–5 months corrected age (CA) and 18–24 months CA, while parental mental health was assessed up to 3–5 months CA.

## Methods

We conducted a single centre randomized controlled trial (RCT), registered at German Clinical Trials Register (DRKS00027996) and approved by the local ethic committee Regensburg (21-2495-202). Recruitment occurred from October 1, 2021, to June 6, 2023, at the University Perinatal Centre Regensburg.

### Participants

Infants born with <32 weeks of gestation or <1500 g were eligible to participate. Exclusion criteria were diagnosis of a genetic syndrome, musculosceletal deformity, palliative care or caregivers with language restriction (German <A1 level). Recruitment was done after birth when infants reached 32 weeks postmenstrual age (PMA) and study enrollment after informed written consent by parents.

### Randomization and intervention

Following baseline assessment, infants were randomized to the intervention or usual care group in a 1:1 ratio. A randomization list was generated by an independent person using the online platform studyrandomizer.com. Randomization was performed by using sequentially numbered opaque envelopes which were opened after inclusion of the patient by the study personal. Twins were allocated to the same group because it was impossible to withhold knowledge or group assignment from the parents and the physical therapists teaching the parents. Both parents and therapists were not blinded to group allocation but asked not to share information with other parents to ensure blinding of therapy. All infants, regardless of group assignment, received usual care which included routine medical care at the NICU, allied health support including physiotherapy e.g. to support drinking for 1–3 times for 30 min per week performed by a physiotherapist.

The intervention consisted of the application of GM-based therapy based on the protocol developed by Soloveichick et al.^18^ (Supplement, Table S1). In deviation to Soloveichick et al.^18^ GM-based therapy was delivered in our trial by parents after training by a pediatric physiotherapists (PT) in order to 1) continue GM-based therapy at home after hospital discharge on a three times daily and cost-effective basis and 2) to strengthen the parent-child-bond and parents´ self-efficacy (Supplement, Table S2). In order to provide an ambient therapy environment for the parents the intervention was accompanied by a selected piece of music composed for premature babies from a jukebox.^19^ To standardize GM-based therapy by parents they received a prerecorded educational video after randomization to the intervention group. Intervention started during hospital stay at 34 weeks PMA and was carried out for 10 weeks, thus until five weeks CA. After discharge home, care and supervision were provided via weekly video conferences.

### Outcomes and follow up

**Fig. 1:**
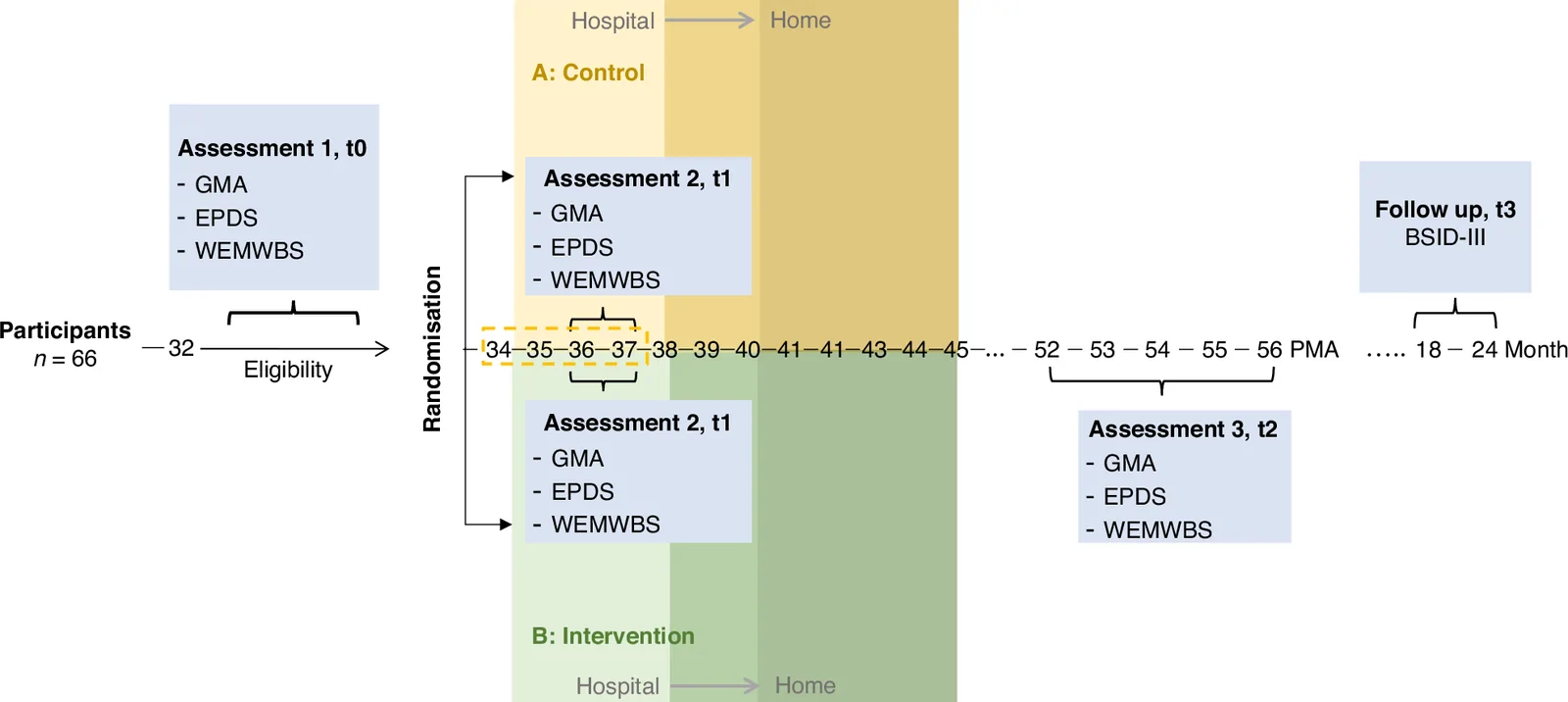
Flow chart of study processes. Schematic overview of participant flow, group allocation, and timing of study assessments. The figure illustrates the assessment time points (t0–t3) and the corresponding outcome measures collected throughout the study. The yellow dashed line indicates time points of neurofilament light chain (NfL) and glial fibrillary acidic protein (GFAP) measurements. n denotes number of participants.

All infants enrolled in the study were assessed on the same schedule (Fig. 1). The parameters of the primary and secondary endpoints were recorded for both study groups at baseline PMA 32–33 weeks (assessment 1, t0), before hospital discharge home at 37–38 weeks PMA (assessment 2, t1) and at 3–5 months CA (assessment 3, t2). At 22-28 months CA all infants were scheduled for a follow-up to assess neurodevelopment by using Bayley Scales of Infant and Toddler Development–Third Edition (BSID-III).

For infants, the primary endpoint was neurodevelopment, assessed at t2 (assessment 3, 3–5 months CA) using the Motor Optimality Score-Revised (MOS-R) and at t3 (follow-up, 18–24 months CA) using the BSID-III.^20,21^ MOS-R rating was conducted by two independent, certified assessors (advanced GMA level) who were blinded to the infants’ clinical histories and intervention groups (MWR and MW). In cases of disagreement, video recordings were jointly reviewed until a consensus on the final score was reached. The BSID-III was administered by an experienced psychologist who was blinded to the intervention.

For parents the primary endpoint was defined as mental health at time t2. Mental health was measured at t0, t1, t2. In accordance with the continuum model,^22^ the well-being of parents was assessed in addition to mental illness by two standardized and validated questionnaires: Edinburgh Postnatal Depression Scale (EPDS) and Warwick-Edinburgh Mental Well-Being Scale.^23,24^

Secondary outcomes included assessing the feasibility of GM-based therapy and examining the association of the biomarkers neurofilament light chain (NfL) and glial fibrillary acidic protein (GFAP) with neurodevelopmental outcomes in children. Feasibility was assessed using adherence data and a post-intervention questionnaire. Adherence was measured through structured parent diaries, which recorded the total number of therapy sessions over 10 weeks (Frequency_TH; range: 0–210; high adherence: 140–210 sessions, medium: 70–140, low: 0–70) and the total therapy duration in minutes (Duration_TH; range: 0–1020 min; high adherence: 700–1020 min, medium: 350–700, low: 0–350). In addition, parents completed a post-intervention questionnaire evaluating the perceived ease or difficulty of implementing the GM-based therapy.^25^ For biomarker analysis, additional blood samples were collected during routine blood draws at 34–37 weeks’ gestation. NfL and GFAP levels were measured using single molecule array (SIMOA) technology as demonstrated previously.^26,27^

### Statistics

#### Sample size calculation

The sample size was based on neurodevelopmental outcomes at 2 years. A similar study^28^ showed a moderate to large effect size (Cohen’s d = 0.8) for BSID-III at 24 months in favor of the motor intervention group. To detect this large effect (*d* = 0.8) with 80% power and a significance level of *α* = 0.05, 26 participants per group were required for analysis. Considering a 20% dropout rate, 33 preterm infants were planned per group. G*Power software (G*Power version 3.1., Heinrich-Heine-University Düsseldorf, Germany) was used for the calculation.

#### Statistical methods

Descriptive statistics were used to present demographic and relevant baseline data for infants and mothers. Categorical variables are presented as absolute number and percentage of the respective category/variable level, metric variables are summarized by mean value with standard deviation or by median (interquartile range (IQR). Analyses of the primary and secondary endpoints were performed using the Full Analysis Set (FAS). The FAS includes all children randomized to the study.

Statistical analyses included univariate tests for initial data exploration, followed by multivariate linear and logistic regressions based on a theoretical model. Independent variables included birth weight, length of hospital stay, and number of complications (BPD, IVH, PHH, ROP).

For MOS-R, additional analyses incorporated poor repertoire (PR) and cramped synchronized (CS) movements at T0, with results reported as regression coefficients (B) and 95% confidence intervals (CIs). MOS-R subscores were analyzed using multinomial logistic regression, with odds ratios (ORs) and 95% confidence intervals (CIs) reported as effect estimates. BSID-III outcomes (cognition, motor skills, language) were analyzed using multiple logistic regression, dichotomized at a cut-off of 85 (<85 vs. ≥85). All secondary outcomes were analyzed using either linear regression models for the MOS-R or logistic regression models for dichotomized outcome parameters, which included fidgety movements at 3–5 months (normal vs. abnormal/absent) and the categories of the BSID-III. Additionally, an ROC analysis was performed for the biomarkers. As adherence was only assessed in the intervention group, it is presented descriptively. All analyses were performed using R Statistical Software (v4.3.2; R Core Team 2021) andall *p*-values are two-sided and were considered statistically significant if p was <0.05.

## Results

**Fig. 2:**
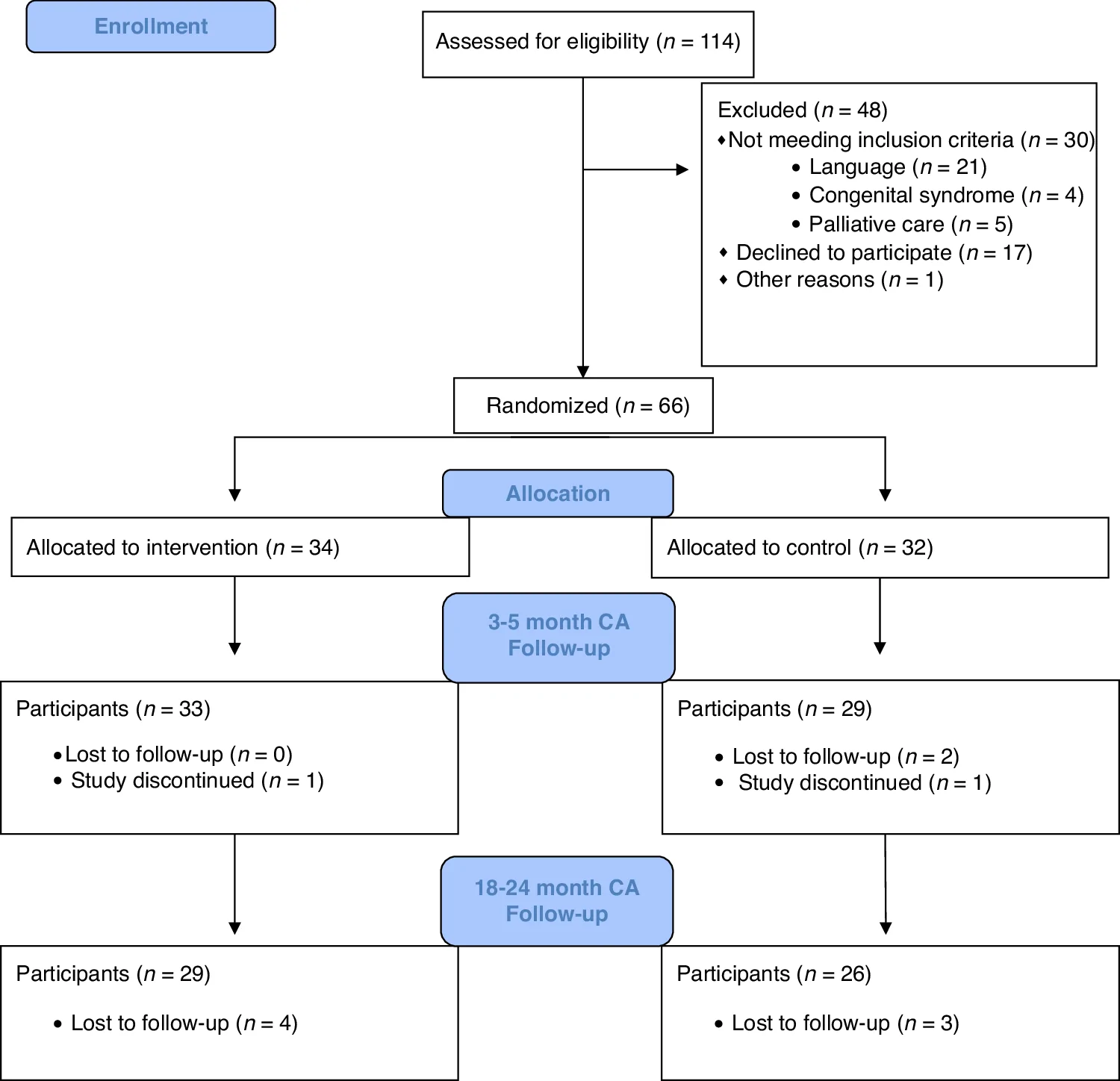
Consort flow diagram of participant enrollment and allocation. Diagram summarizing participant enrollment, allocation, follow-up, and analysis according to CONSORT guidelines. *n* denotes number of participants; CA corrected age.

Between October 1, 2021 and June 6, 2023 114 parents were approached to participate in the GeMo-Support study, 48 were excluded, and 66 randomized to either the intervention group or the control group (CONSORT flow chart, Fig. 2). In detail, 32 children (including 3 pairs of twins) and their 29 parents were randomized to the control group, and 34 children (including 5 pairs of twins) and their 29 parents were randomized to the intervention group. Characteristics are shown in Table 1. A total of four children were lost to follow-up at 3–5 months CA and a further seven at 18–24 months CA.

**Table 1:** Characteristics of infants and their mothers.

Characteristics No.(%)	Overall	Control	Intervention
Mothers^a^	*n* = 58	*n* = 29	*n* = 29
Age, median (IQR)	32.0 (29.0, 35.0)	32.0 (29.0, 35.0)	32.0 (28.0, 35.0)
Socioeconomic status, median (IQR)	14.8 (12.7, 17.8)	13.7 (12.7, 17.1)	15.4 (13.9, 17.8)
German speaking	48 (83%)	24 (83%)	24 (83%)
Cesarean section	41 (71%)	21 (72%)	20 (69%)
Preeclampsia	20 (34%)	8 (28%)	12 (41%)
Diabetes gestational	3 (5.2%)	2 (6.9%)	1 (3.4%)
Maternal infection	11 (19%)	4 (14%)	7 (24%)
Placental insufficiency	14 (24%)	5 (17%)	9 (31%)
PPROM	16 (28%)	10 (34%)	6 (21%)
Preterm labour	20 (34%)	10 (34%)	10 (34%)
Pathological CTG	16 (28%)	9 (31%)	7 (24%)
Time between birth and first antenatal steroids, median (IQR), *d*	3.0 (1.0, 11.5)	3.0 (1.0, 12.0)	3.0 (1.0, 9.0)
Pregnancy first	36 (62%)	20 (69%)	16 (55%)
Parturition first	40 (69%)	21 (72%)	19 (66%)
Infants*	*n* = 66	*n* = 32	*n* = 34
Gestational age, median (IQR), wk	29.0 (26.3, 30.0)	30.0 (27.0, 30.0)	28.5 (26.0, 30.0)
Birth weight, median (IQR), *g*	1105 (885, 1408)	1243 (919, 1623)	1035 (853, 1230)
Sex			
Male	37 (56%)	15 (47%)	22 (65%)
Female	29 (44%)	17 (53%)	12 (35%)
Singelton birth	48 (73%)	25 (78%)	23 (68%)
Umbilical cord PH, median (IQR)	7.30 (7.26, 7.34)	7.32 (7.27, 7.34)	7.30 (7.25, 7.35)
Apgar score, median (IQR)			
At 5 min	8.00 (7.00, 8.00)	8.00 (7.00, 9.00)	8.0 (7.00, 8.00)
At 10 min	9.00 (8.00, 9.00)	9.00 (8.8, 9.00)	9.00 (8.00, 9.00)
Hospitalization, median (IQR), *d*	51.5 (42.0, 75.0)	45.5 (38.0, 71.3)	65.0 (43.0, 85.8)
Discharge with medical supplies	10 (15%)	4 (13%)	6 (18%)
Exclusive nutrition with mother milk at discharge	38 (58%)	18 (56%)	20 (59%)
Severe Complications			
BPD (moderate to severe)	8 (12%)	2 (6.3%)	6 (18%)
IVH > grade 2	3 (4.5%)	2 (6.3%)	1 (2.9%)
PHH requiring shunt therapy	2 (3.0%)	1 (3.1%)	1 (2.9%)
ROP > grade 2	3 (4.5%)	0 (0%)	3 (8.8%)
NEC/FIP/Ileus requiring operation	5 (7.6%)	4 (13%)	1 (2.9%)
GM-Preterm			
CS	3 (4,5%)	0 (0%)	3 (8.8%)
N	22 (33%)	13 (41%)	9 (26%)
PR	41 (62%)	19 (59%)	22 (65%)
GM-Writhing			
CS	4 (6%)	1 (3%)	3 (8.8%)
N	15 (23%)	6 (19%)	9 (26%)
PR	45 (68%)	24 (75%)	21 (62%)

*IQR* Interquartile Range, *BPD* bronchopulmonary disease, *GM* General Movements, *IVH* intraventricular hemorrhage, *NEC/FIP* necrotizing enterocolitis/ focal intestinal perforation, *PHH* posthemorrhagic hydrocephalus, *PPROM* preterm premature rupture of membranes (>18 h before delivery), *ROP* retinopathy of prematurity, *d* days, *g* gramm, *wk* weeks, *CS* cramped synchronized, *N* normal movements, *PR* poor repertoire.

^a^please note that the number of mothers and infants is different due to the inclusion of twins.

**Fig. 3:**
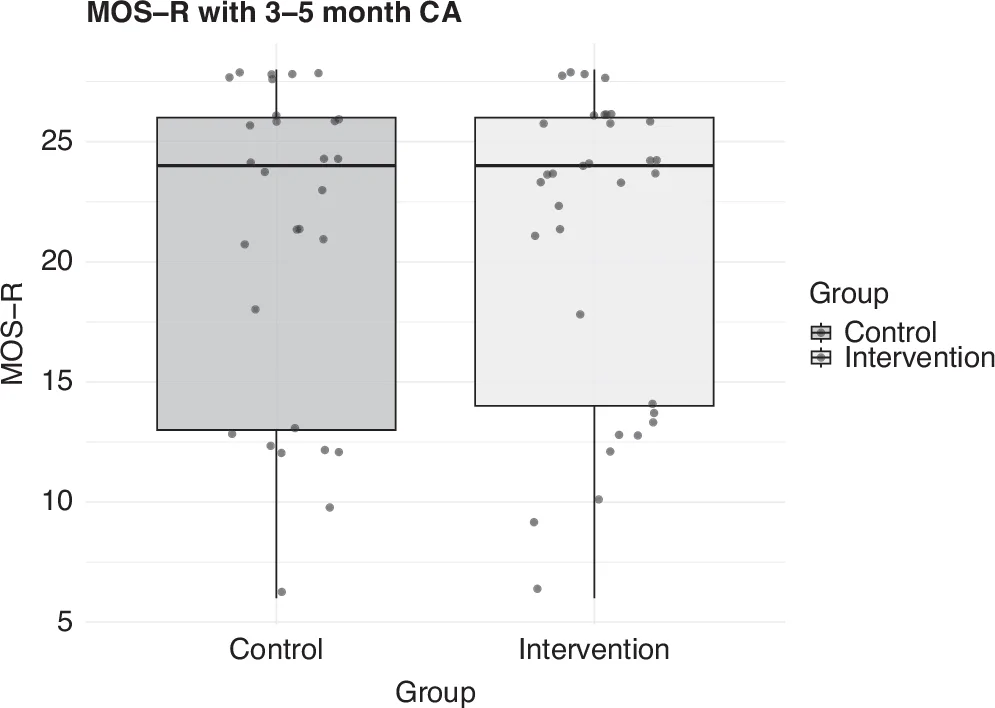
MOS-R scores at time t2 (3–5 month corrected age). Boxplots of MOS-R scores at follow-up (t2) in the control and intervention groups. Boxes indicate the interquartile range with the median; dots represent individual participants. CA corrected age.

**Fig. 4:**
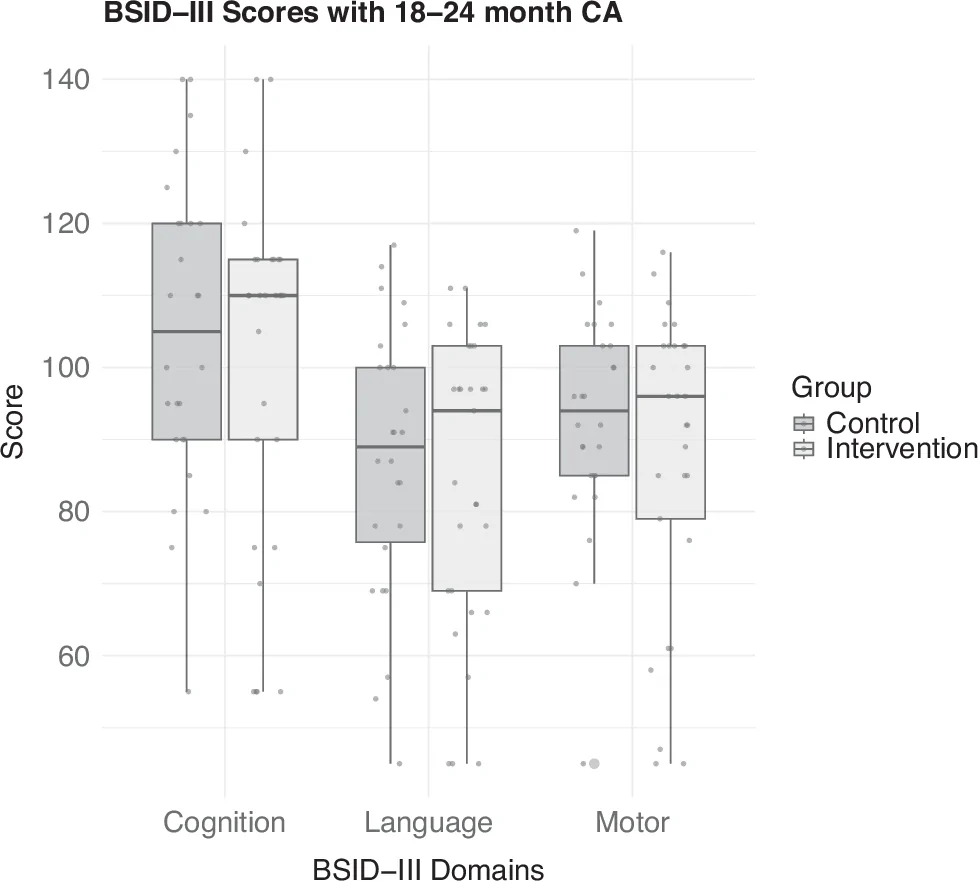
BSID-III scores at time t3(18–24 months corrected age). Boxplots of cognitive, language, and motor BSID-III composite scores at follow-up (t3) in the control and intervention groups. Boxes indicate the interquartile range with the median; dots represent individual participants. CA corrected age.

**Fig. 5:**
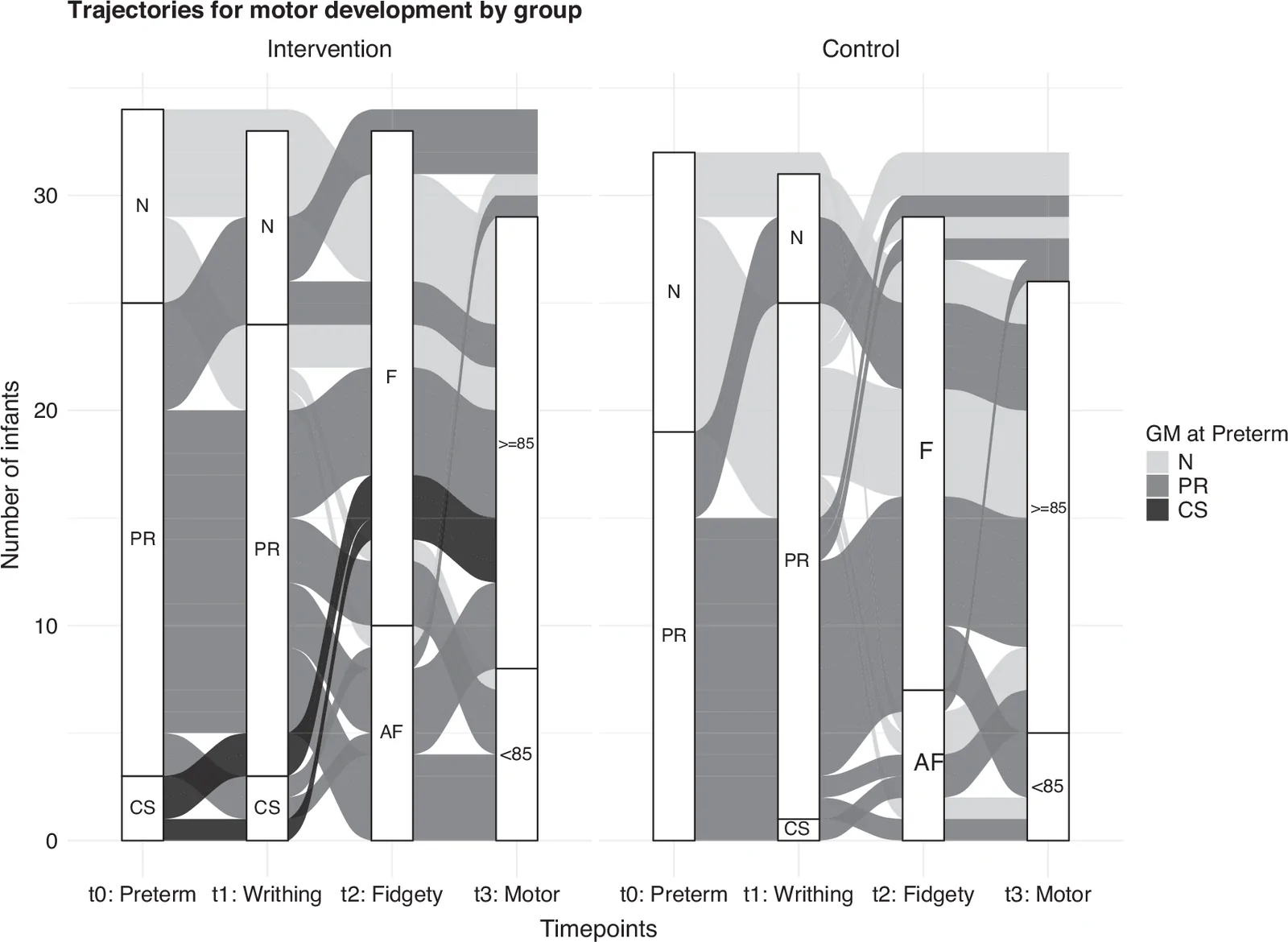
Trajectories of motor development by group. Alluvial plot illustrating trajectories of motor development across assessment time points (t0–t3) in the intervention and control groups. Bar heights represent the number of infants in each category, and flows indicate transitions between categories over time. t0, preterm; t1, writhing movements; t2, fidgety movements; t3, motor outcome (BSID-III). N normal, PR poor repertoire, CS cramped-synchronized, F fidgety, AF absent/abnormal fidgety.

Primary endpoints: There was no difference in the primary outcome MOS-R at 3–5 months CA and BSID-III at 18–24 months CA between the intervention and control groups in preterm infants. The MOS-R scores were 24.0 (IQR: 4.0–26.0) vs. 24.0 (IQR: 13.0–26.0) (*p* = 0.986), with no significant regression results (*B* = 0.96, 95%-CI: −2.37, 4.14, *p* = 0.583) (Fig. 3). A multinominal analysis of the individual categories of the MOS-R showed no significant differences between the groups (Table 2). BSID-III median (IQR) scores were in the intervention vs. control group for cognition 110 (90–115) vs. 105 (90–120), for language 94 (69–103) vs. 89 (75.75–100), and for motor skills 96 (79–103) vs. 94 (85–103), respectively, with no significant differences between groups (cognition OR = 0.71, *p* = 0.704; language OR = 0.90, *p* = 0.870; motor skills OR = 0.90, *p* = 0.870) (Fig. 4, Table 3). Subanalysis revealed no differences in short- and long-term neurodevelopmental outcome (MOS-R and BSID-III) in favor of the intervention when analyzing subgroups of children with CS, PR, or normal movement at t0 or t1. Interestingly, all three infants displaying CS movements in the intervention group before treatment showed good neurodevelopment (Fig. 5).

**Table 2:** MOS-R with Subcategories at 3–5 month corrected age.

		Overall *n* = 62	Control *n* = 29	Intervention *n* = 33	Intervention vs. Control^a^
Subscore	Score				OR 95% CI; *p*-value
Fidgety	1	8 (13%)	4 (14%)	4 (12%)	
	4	9 (15%)	3 (10%)	6 (18%)	2.00 (0.17, 3.35); 0.488
	12	45 (73%)	22 (76%)	23 (70%)	1.05 (1.90, 16.00); 0.954
Movement Pattern	1	5 (8.1%)	1 (3.4%)	4 (12%)	
	2	6 (9.7%)	6 (21%)	0 (0%)	0.000018 (1.58e-46, 2.04e36); 0.821
	4	51 (82%)	22 (76%)	29 (88%)	0.33 (0.03, 3.16); 0.336
Age Adequate Movement	1	10 (16%)	6 (21%)	4 (12%)	
	2	35 (56%)	15 (52%)	20 (61%)	2.00 (0.478, 8.37); 0.343
	4	17 (27%)	8 (28%)	9 (27%)	1.69 (0.346, 8.22); 0.517
Postural Pattern	1	11 (18%)	5 (17%)	6 (18%)	
	2	8 (13%)	4 (14%)	4 (12%)	0.833 (0.134, 5.17); 0.845
	4	43 (69%)	20 (69%)	23 (70%)	0.958 (0.254, 3.62); 0.950
Movement Character	2	44 (71%)	20 (69%)	24 (73%)	
	4	18 (29%)	9 (31%)	9 (27%)	0.834 (0.278, 2.50); 0.745

^a^Multinominal regression using intervention as predictor.

**Table 3:** BSID-III Domains at 18–24 month corrected age.

BSID-III Domains	Overall *n* = 55			Control *n* = 26			Intervention *n* = 29			Intervention vs. Control unadjusted	Intervention vs. Control^*^
	Median (IQR)	Mean (SD)	<85 *n* (%)	Median (IQR)	Mean (SD)	<85 *n* (%)	Median (IQR)	Mean (SD)	<85 *n* (%)	U-value, *p*-value	OR (95%CI); *p*-value
Cognition	110 (90–115)	102.64 (23.13)	11 (20)	105 (90–120)	104.81 (21.47)	4 (15.38)	110 (90–115)	100.69 (24.74)	7 (24.14)	406, *p* = 0.629	0.71 (0.11, 4.07), *p* = 0.704
Language	91 (69–101.5)	85.73 (19.75)	25 (45.45)	89 (75.75–100)	87.04 (18.93)	11 (42.31)	94 (69–103)	84.55 (20.72)	14 (48.28)	396, *p* = 0.747	0.90 (0.26–3.04), *p* = 0.870
Expressiv	9 (6–11)	8.25 (3.67)		9 (7–11)	8.50 (3.25)		9 (5–11)	8.03 (4.06)		395, *p* = 0.765	
Receptiv	7 (6–9)	7.16 (3.2)		7 (6–9.75)	7.38 (3.29)		8 (6–9)	6.97 (3.12)		399,4, *p* = 0.708	
Motor	96 (85–103)	90.3 (18.36)	13 (23.34)	94 (85–103)	92.85 (15.19)	5 (19.23)	96 (79–103)	88.03 (20.81)	8 (27.59)	402, *p* = 0.678	0.80 (0.15–3.78), *p* = 0.774
Fine Motor	9 (7.5 –10)	8.6 (3.15)		9 (8–10.75)	9.12 (2.75)		9 (7–10)	8.14 (3.45)		427, *p* = 0.399	
Gross Motor	9 (8–11)	8.58 (3.14)		9 (8–11)	8.92 (2.58)		9 (7–11)	8.28 (3.59)		384,5, *p* = 0.902	

^a^multiple linear regression model adjusted for baseline and known confounders.

**Fig. 6:**
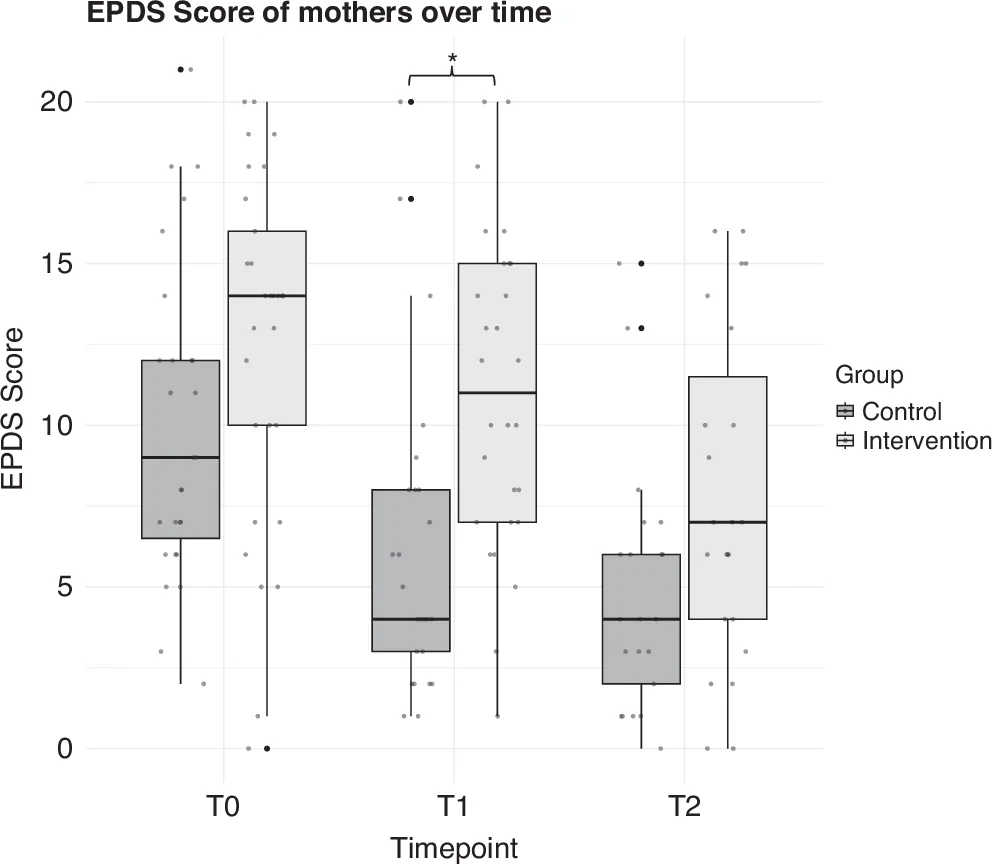
Maternal EPDS score over time. Boxplots of Edinburgh Postnatal Depression Scale (EPDS) scores assessed at T0, T1, and T2 in the control and intervention groups. Boxes indicate the interquartile range with the median; dots represent individual participants. Asterisk indicates a statistically significant between-group difference. EPDS Edinburgh Postnatal Depression Scale.

For parents, the primary outcome also showed no significant difference at 3–5 months CA. No significant difference in mental health (EPDS) was observed (Table 4). Maternal mental health at t1 (assessment 2) was lower in the intervention group (EPDS: *B* = 3.34, 95%-CI: 0.62, 6.05, *p* = 0.017), but this association was no longer present at t2 (assessment 3) (EPDS: *B* = 0.62, 95%-CI: -2.03, 3.27, *p* = 0.640) (Fig. 6). Overall, maternal mental health, independent of intervention, at 3–5 months negatively influenced the likelihood of a positive MOS-R outcome (OR = 0.84, *p* = 0.026), but no impact was observed on neuromotor development at 18-24 months.

**Table 4:** Outcome for parents with 3–5 months CA.

	Overall median (IQR); *n*	Control median (IQR); *n*	Intervention median (IQR); *n*	Intervention vs. Control unadjusted (r (95%CI), *p*-value)	Intervention vs. Control^a^ (B (95%CI) *p*-value)
Mother					
EPDS – T0	12.0 (7.0–15.0); 56	9.0 (6.5–12.0); 27	14.0 (10.0–16.0); 29		
EPDS – T1	8.0 (4.0–13.0); 53	4.0 (3.0–8.0); 25	11.0 (7.0–15.0); 28	3.66 (1.04, 6.28), *p* = 0.007	3.34 (0.62,6.05), *p* = 0.017
EPDS – T2	6.0 (3.0–8.3); 44	4.0 (2.0–6.0); 21	7.0 (4.0–11.5); 23	1.74 (−0.96, 4.44), *p* = 0.199	0.62 (−2.03, 3.27), *p* = 0.640
WEMWS – T0	53.0 (45.8–56.3); 56	54.0 (49.0–61.5); 27	49.0 (42.0–55.0); 29		
WEMWS – T1	54.0 (50.0–60.0); 53	57.0 (52.0–61.0); 25	52.0 (47.3–57.0); 28	−2.401 (−6.13; 1.33), *p* = 0.202	−1.92 (−5.90,2.06), *p* = 0.337
WEMWS – T2	56.0 (52.0–63.0); 45	59.0 (52.5–63.0); 22	56.0 (52.5–60.5); 23	−1.618 (−5.56, 2.33), *p* = 0.413	−0.73 (−5.04, 3.58), *p* = 0.733
Father					
EPDS – T0	6.0 (4.0–11.0); 51	6.5 (2.5–9.0); 24	6.0 (5.0–11.0); 27		
EPDS – T1	7.0 (5.0–9.0); 46	7.0 (5.0–9.0); 19	6.0 (5.0–9.0); 27	−1.20 (–3.25, 0.85), *p* = 0.244	−1.75 (−4.03, 0.53), *p* = 0.128
EPDS – T2	3.0 (1.0–6.0); 38	3.0 (1.0–5.5); 19	3.0 (1.5–6.5); 19	−0.39 (–2.89, 2.12), *p* = 0.743	−1.05 (−4.01, 1.99), *p* = 0.485
WEMWS – T0	56.0 (50.0–60.0); 50	56.0 (52.0–62.5); 23	53.0 (49.5–58.5); 27		
WEMWS – T1	56.5 (52.0–60.0); 46	58.0 (55.0–64.0); 19	55.0 (52.0–59.0); 27	–1.76 (–5.22, 1.69), *p* = 0.309	1.62 (−2.79, 6.04), *p* = 0.46
WEMWS – T2	55.0 (51.3–61.0); 38	55.0 (52.0–63.0); 19	55.0 (51.0–60.0); 19	0.62 (2.96, 4.19), *p* = 0.729	1.62 (–2.79, 6.04), *p* = 0.459

^a^multiple linear regression model adjusted for baseline and known confounders.

*EPDS* Edinburgh Postnatal Depression Scale, *WEMWS* Warwick-Edinburgh Mental Well-Being Scale.

Secondary Endpoints: For 33 of the 34 children in the intervention group, we received parental reports on therapy compliance. The median frequency and duration of therapy were 64 sessions (IQR 50-95) and 350 minutes (IQR 270-585). Only six participants met high compliance criteria. Reasons for not performing the intervention or spending less time were related to infant behavioral state, care tasks, absence of parents, or other therapies. No significant correlation was found between therapy frequency/duration and the MOS-R score. However, therapy frequency had a long-term effect on BSID-III language development in univariate analysis with better outcome for those with more therapy (0.259, 95%-CI [0.058, 0.460], *p* = 0.014). Further, no statistically significant correlation was found between the mother’s mental health and adherence (EPDS: −0.07, 95%-CI: −2.48, 2.35, *p* = 0.956). The provided answers to the therapy evaluation questionnaire showed that almost all parents felt very well supported to conduct the therapy and that they could actively support their child with the therapy, but found it difficult to practice several times a day.

Analysis of biomarkers showed no significant differences between the intervention and control group for NfL (*p* = 0.553) and GFAP concentrations (*p* = 0.080). Univariate analysis showed a significant difference in Fidgety Movements (*p* = 0.04) with higher concentrations in infants with abnormal/absent movements for NfL (Median 10.02 pg/ml, IQR: 8.57–14) and GFAP (Median 315.34 pg/ml, IQR: 252.11–396.72 as compared to infants with normal movements for NfL (Median 6.85 pg/ml, IQR: 6.21–10.54 and GFAP (Median 277.49 pg/ml, IQR: 192.94–385.38). At 18-24 months, BSID-III cognition scores were significantly associated with NfL (OR = 0.78, 95%-CI [0.62, 0.93], *p* = 0.011) and GFAP (OR = 0.99, 95%-CI [0.98, 1.00], *p* = 0.012) and BSID-III motor scores (NfL: OR = 0.89, 95%-CI [0.69, 1.11], *p* = 0.044; GFAP: OR = 0.99, 95%-CI [0.99, 0.998], *p* = 0.027). However, in multivariate analysis correcting for birth weight, length of hospital stay, and number of complications (BPD, IVH, PHH, ROP) no significance was observed. The ROC analysis is presented in Table 5.

**Table 5:** ROC-Analysis of biomarkers NfL and GFAP

Outcome	Biomarker	Cutoff	Sensitivity	Specificity	AUC
Fidgety abnormal/absent vs normal	NfL	8.87 pg/ml	0.657	1.000	0.789
BSID-III Motor domain <85 vs. ≥85	NfL	8.23 pg/ml	0.588	1.000	0.746
	GFAP	306.49 pg/ml	0.735	0.909	0.802
BSID-III Cognition domain <85 vs. ≥85	NfL	8.87 pg/ml	0.657	1.000	0.789
	GFAP	306.49 pg/ml	0.714	0.900	0.834

## Discussion

Normal neurological development during early childhood is critically dependent on appropriate motor development.^14^ Preterm infants with impaired motor function often face significant challenges in achieving key neurodevelopmental milestones.^15^ Early motor interventions that simulate age-specific GM patterns may enhance neurodevelopmental outcomes in this high-risk population.^18^

To evaluate this hypothesis, we conducted an RCT comparing GM-based therapy—administered by trained parents three times daily over a 10-week period—with standard care in very preterm infants, irrespective of cerebral comorbidities. The intervention commenced during NICU stay at 34 weeks PMA and continued for 10 weeks post-discharge, with ongoing support provided to parents via telemedicine by pediatric physiotherapists.

The intervention was demonstrated to be feasible and well-tolerated. However, no significant differences were observed between the intervention and control groups in neurodevelopmental outcomes up to two years of age, nor in measures of parental mental health. Notably, all three infants in the intervention group who exhibited poor neuromotor function at baseline showed favorable neurodevelopmental outcomes at follow-up.

Motivated by the encouraging results of an initial GM-based case study that translated Prechtl’s vision into a therapeutic framework,^18^ we implemented the same intervention across a broader cohort of very preterm infants, irrespective of the presence of cerebral comorbidities. Our hypothesis—that all very preterm infants might benefit from targeted motor therapy regardless of neurological status—was not supported by the findings. Neither infants exhibiting normal GMs nor those with abnormal GMs (poor repertoire, PR) at discharge demonstrated significant neuromotor improvement following the GM-based intervention. Notably, only three infants in the study displayed pathological GMs (characterized by cramped-synchronized, CS movements), all of whom were randomized to the intervention group. Given the small number of such cases, the effectiveness of the intervention in this specific subgroup remains inconclusive and warrants further investigation.

Another key distinction between our study and that of Soloveichik et al.,^18^ which may have influenced the effectiveness of the intervention, was the level of compliance among therapy providers. In the case study by Soloveichik et al.,^18^ the intervention was delivered with high intensity—five sessions per day, each lasting up to 20 min, over a 10-week period. This frequency and duration of therapy, however, proved unfeasible in our clinical setting. Moreover, the compliance in our study was relatively low. Only 63.6% of parents in the intervention group achieved medium to high adherence, defined as 140–210 sessions totalling 700–1020 min over the 10-week period. Several factors contributed to this reduced compliance, including the infant’s behavioral state, concurrent caregiving demands, parental absence, and scheduling conflicts with other therapies. Similar challenges have been reported in previous motor intervention studies, where—despite high parental acceptance of the intervention—many found it too demanding to consistently administer therapy five days per week.^10^ Importantly, GM–based intervention was conducted in preterm infants of comparable gestational age, suggesting that differences in adherence and intervention intensity, rather than population characteristics, are the most plausible explanation for the divergent results.

There is substantial evidence that early interventions are most effective in improving neurodevelopmental outcomes when delivered by parents.^13,29^ In our study, however, we observed a transient negative impact of the intervention on maternal mental health at the time of discharge, an effect that was no longer present at 3–5 months corrected age (CA). We speculate that this temporary decline in maternal mental health may be attributable to increased stress associated with participation in the study and the demands of delivering the therapy at the prescribed frequency. Prior research has demonstrated a strong correlation between elevated maternal stress levels and symptoms of depression.^30^

This interpretation is further supported by qualitative feedback from the therapy evaluation. While nearly all participating parents reported feeling empowered to support their child through the intervention, many expressed difficulties in maintaining the required frequency of practice multiple times per day. It is important to note that, despite the statistically significant difference in maternal mental health scores between groups at discharge, the intervention group showed subsequent improvement. By follow-up, their scores fell within a range indicative of moderate well-being and only a moderate risk of depressive symptoms.

Since previous studies on motor interventions have employed various heterogeneous outcome parameters, comparing their results to our data is challenging. Recent systematic reviews and meta-analyses on NICU-based intervention programs for motor outcomes demonstrate sustained effects up to 24 months.^13,31^ However, studies within physiotherapy primarily show effects in the short term up to 4 month, focusing on clinical parameters, neuromuscular and neurological signs, motor development, and behavioral state.^11,12,32^ Whereas early developmental interventions have been shown to improve neurodevelopmental outcome for preterm infants in infancy,^33^ it is unclear weather motor interventions per se initiated during NICU stay improve neurodevelopmental outcome because of methodological heterogeneity and a lack of long-term outcomes.^34,35^

The biomarker analysis conducted within the study produced encouraging findings. A significant association was identified between NfL concentrations and the presence or absence of fidgety movements. Furthermore, at 18–24 months corrected age, both NfL and GFAP levels were significantly correlated with motor and cognitive outcomes, demonstrating good sensitivity and specificity. These results suggest that NfL and GFAP hold potential as prognostic biomarkers in very preterm infants and support previous findings of our group and others.^36,37^ In particular, they may aid in the early identification of children at risk for neurodevelopmental impairments, thereby facilitating the timely initiation of targeted interventions and structured follow-up programs. Although these associations were attenuated and no longer significant in multivariable analyses, the consistent direction of effects and their biological plausibility underscore that NfL and GFAP remain promising markers for early risk stratification. These findings, therefore, provide valuable preliminary evidence that warrants confirmation in larger, adequately powered studies.

Several limitations must be acknowledged in the interpretation of our findings. Firstly, the sample size was determined based on the assumption of large effect sizes, which likely resulted in insufficient power to detect smaller effects. Additionally, our study included a heterogeneous population of very preterm infants, irrespective of the presence of cerebral comorbidities. As highlighted by Hutchon et al., a “one-size-fits-all” approach is not effective for high-risk infants and their families; rather, interventions should be individualized to meet the specific needs and circumstances of each child and family.^6^

Consequently, future research should place greater emphasis on the careful selection of inclusion and exclusion criteria for intervention groups. This should involve consideration of the most appropriate target populations, e.g. preterm infants with early signs of poor neuromotor function, optimal timing for intervention, and the most effective modes of delivery. Another limitation is the inability to blind parents to group allocation, which raises the possibility of a spillover effect favoring the control group. To mitigate this risk, parents in the intervention group were explicitly instructed not to disclose or discuss the details of the intervention with other parents in the NICU.

## Conclusion

This report of a randomized clinical feasibility trial demonstrates that parent-delivered GM-based therapy can be implemented in very preterm infants. There was no evidence that administration by parents did improve neurodevelopment of their infants or mental health of parents. Relatively poor parental compliance and the inclusion of patients without cerebral compromise may have diluted any effect. Larger trials are needed to assess efficacy.

## Supplementary information

Supplement-New Version

## Data Availability

The datasets used and/or analysed during the current study are available from the corresponding author on reasonable request.
