# Supplementary material for: General movement based therapy to support neurodevelopment of preterm infants: a randomized clinical trial
Source: Pediatr Res. 2026 Jan 9;100(3):1291–300. doi: 10.1038/s41390-025-04734-2 (PMC13672066; doi:10.1038/s41390-025-04734-2)
Supplement: Supplementary file 1 [file 41390_2025_4734_MOESM1_ESM.pdf]

Supplement

Supplement Table S1: General description of the intervention

General preparation	Preparation of the environment and the child for therapy in accordance with the initial situation.
Position of infant for the intervention	Supine position on bed or thigh, feets facing towards the therapist/parents, optional nesting (in fussy or extremely hypotonic infants)
Course of intervention	Infants showing poor repertoire (PR), cramped synchronized (CS) or chaotic movements (Ch) are gently maneuvered in each movement episode via the extremities towards normal GM imitating sequences. Movements are transformed into calm and fluid movements and attempts are made to overlay them with variable movements. Infants showing normal GM are softly guided through the movements
Behavioural state during intervention	"State of arousal level" three (eyes open, no movements) or four (eyes open, large movements) according to Prechtl's states.
Pausing/Stopping rules	If the infant shows any of the following signs which are interpreted as expressions of stress or discomfort: makes faces, changes skin colour, irregular respiration, undesired changes in muscle tone, uncontrolled movements or continual changes in the state of arousal level
Performance time	Adjusted to infant's daily rhythm, half an hour before a meal, between two meals or any time when the child is in a state of arousal level three or four.

Supplement Table S2: Schedule for the instruction of parents

Steps	Teaching Elements	Strategies	Timeline
Step 1: Familiarization	Convey the idea of general movements	Informational video	1-3 sessions
	Explain different aspects of variability: amplitude of movements, different speeds, range of motion, fluid start and stop of movements, flow of movement	Explanation during observation of the infant/GMA video	
	Getting to know the individual child's movements		
	Showing different and correct position of the child during exercises	Demonstration<br>Supervised positioning	
	Teaching handling techniques to parents: guiding and sliding with maximal contact	Demonstration<br>Supervised handling	
Step 2: Experience	Let parents practice and encourage them to incorporate the exercises	Tips for best timepoints to practice<br>Offer time alone to practice but support at every time point	After the familiarization and new input for 1-3 days.
Step 3: Supervision	Work on the correct start of initiating the exercises	Demonstration/Observation/Video analysis of their child	Individual schedule depending on the skill of the parents
	Point out different aspects of variability	Give ideas of possible movements<br>Demonstration/Video analysis<br>General Movement Optimality Score	
	Teach behavioral signs to stop exercises or pause	Training in how to observe and interpret their infant's behaviors and physiological signs of pleasure and stress.	
	Addressing difficulties: Hypotonic/hypertonic	Ask for difficulties and give advice how to handle these.	
	Evaluate the child's movements through video	General Movement Optimality Score	
